# Nitrite as a causal factor for nitrate‐dependent anaerobic corrosion of metallic iron induced by *Prolixibacter* strains

**DOI:** 10.1002/mbo3.1225

**Published:** 2021-08-16

**Authors:** Takao Iino, Nobuaki Shono, Kimio Ito, Ryuhei Nakamura, Kazuo Sueoka, Shigeaki Harayama, Moriya Ohkuma

**Affiliations:** ^1^ Japan Collection of Microorganisms (JCM) RIKEN BioResource Research Center (RIKEN‐BRC) Tsukuba Japan; ^2^ Biofunctional Catalyst Research Team Center for Sustainable Resource Science, RIKEN Wako Japan; ^3^ Resource and Process Solution Division Mineral Resources Department Nippon Steel Technology Co., Ltd. Futtsu Japan; ^4^ Earth‐Life Science Institute (ELSI) Tokyo Institute of Technology Meguro‐ku Japan; ^5^ Environment Research Laboratory Advanced Technology Research Laboratories Nippon Steel Co., Ltd. Futtsu Japan; ^6^ Department of Biological Sciences Faculty of Science and Engineering Chuo University Bunkyo‐ku Japan; ^7^ Present address: Chitose Laboratory Corp. Biotechnology Research Center Kawasaki Japan

**Keywords:** iron corrosion, microbially influenced corrosion, nitrate reduction, *Prolixibacter*

## Abstract

Microbially influenced corrosion (MIC) may contribute significantly to overall corrosion risks, especially in the gas and petroleum industries. In this study, we isolated four *Prolixibacter* strains, which belong to the phylum *Bacteroidetes*, and examined their nitrate respiration‐ and Fe^0^‐corroding activities, together with two previously isolated *Prolixibacter* strains. Four of the six *Prolixibacter* strains reduced nitrate under anaerobic conditions, while the other two strains did not. The anaerobic growth of the four nitrate‐reducing strains was enhanced by nitrate, which was not observed in the two strains unable to reduce nitrate. When the nitrate‐reducing strains were grown anaerobically in the presence of Fe^0^ or carbon steel, the corrosion of the materials was enhanced by more than 20‐fold compared to that in aseptic controls. This enhancement was not observed in cultures of the strains unable to reduce nitrate. The oxidation of Fe^0^ in the anaerobic cultures of nitrate‐reducing strains occurred concomitantly with the formation of nitrite. Since nitrite chemically oxidized Fe^0^ under anaerobic and aseptic conditions, the corrosion of Fe^0^‐ and carbon steel by the nitrate‐reducing *Prolixibacter* strains was deduced to be mainly enhanced via the biological reduction of nitrate to nitrite, followed by the chemical oxidation of Fe^0^ to Fe^2+^ and Fe^3+^ coupled to the reduction of nitrite.

## Graphical abstract

Anaerobic corrosion by *Prolixibacter*.

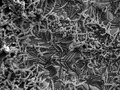

## 1 INTRODUCTION

It has long been recognized that certain microorganisms accelerate the corrosion of carbon‐ and stainless steel. This phenomenon is referred to as microbially influenced corrosion (MIC) (Javaherdashti, 2008). Under aerobic conditions, microorganisms can promote or inhibit the corrosion of metallic iron (Fe^0^) through oxygen respiration (Zarasvand & Rai, 2014). A specific group of sulfate‐reducing bacteria (SRB) accelerates Fe^0^ corrosion in anaerobic environments (Enning & Garrelfs, 2014), with recent evidence indicating that the direct electron transfer from Fe^0^ to SRB via extracellular electron transfer is mediated by outer‐membrane cytochromes (Beese‐Vasbender et al., 2015; Deng et al., 2018; Dinh et al., 2004; Venzlaff et al., 2013). Some hydrogenotrophic methanogens that reduce carbon dioxide to methane (CO_2_ respiration) are capable of causing MIC on Fe^0^ (Daniels et al., 1987; Mori et al., 2010; Uchiyama et al., 2010) by secreting specific [NiFe] hydrogenase, thereby catalyzing the oxidation of Fe^0^ to ferrous ion: Fe^0^ + 2H^+^ → Fe^2+^ + H_2_ (Tsurumaru et al., 2018).

In addition to SRB and methanogens, three facultatively anaerobic nitrate‐reducing bacteria (NRB), namely *Paracoccus denitrificans*, *Bacillus licheniformis*, and *Pseudomonas aeruginosa*, have been found to enhance the corrosion of carbon steel in the presence of nitrate (Ginner et al., 2004; Jia et al., 2017; Till et al., 1998; Xu et al., 2013). *Shewanella oneidensis* MR‐1, notable for its diverse respiratory capabilities also stimulated Fe^0^ corrosion via nitrate respiration (De Windt et al., 2003; Miller II et al., 2018).

Seawater is often injected into oil‐ and gas reservoirs for enhanced oil and gas recovery. However, the high sulfate content of seawater can lead to corrosion and reservoir souring. To mitigate it, nitrate injection has been introduced during the last decades to promote the growth of NRB which can outcompete SRB for carbon sources (Veshareh & Nick, 2019). We previously isolated an NRB from an oil field in Northern Japan, that corroded Fe^0^ under anaerobic conditions (Iino, Ito, et al., 2015). This strain was classified as *Prolixibacter denitrificans* sp. nov., and is the first Fe^0^‐corroding NRB belonging to the phylum *Bacteroidetes* (Iino, Ito, et al., 2015). However, only two strains, *Prolixibacter bellariivorans* strain F2^T^ (Holmes et al., 2007) and *P*. *denitrificans* MIC1‐1^T^ (Iino, Ito, et al., 2015), have been isolated thus far. To explore the potential environmental functions and diversity of *Prolixibacter* strains, we were interested in isolating more *Prolixibacter* strains and characterizing their ability to corrode Fe^0^ under anoxic conditions. In this study, four strains belonging to the genus *Prolixibacter* were newly isolated from oil‐ and gas fields and crude oil storage tanks in Japan. We found that some but not all the *Prolixibacter* strains enhanced the corrosion of Fe^0^. The basis for the phenotypic differentiation between the *Prolixibacter* strains was also investigated.

## 2 MATERIALS AND METHODS

### 2.1 Bacterial strains and culture conditions

Artificial seawater (Sw) medium was composed of (L^−1^): 0.15 g NH_4_Cl, 0.2 g KH_2_PO_4_, 0.5 g KCl, 18.0 g NaCl, 2.6 g MgCl_2_·6H_2_O, 0.15 g CaCl_2_·2H_2_O, 2.5 g NaHCO_3_, 1% (vol/vol) of filter‐sterilized vitamin solution, as described by Wolin et al. (1963), and 1.0 ml of a trace element solution, as used by Touzel and Albagnac (1983), lacking NaCl and supplemented with 4.0 mg/L of Na_2_SeO_3_·5H_2_O. Sulfate/Pyruvate/Yeast‐extract/Seawater (SPYSw) medium was prepared by adding 10 mM sodium sulfate, 10 mM sodium pyruvate, and 0.01% (wt/vol) yeast extract (Bacto Yeast Extract, Becton Dickinson) to Sw medium. Yeast‐extract/Polypeptone/Seawater (YPSw) medium was prepared by adding 0.2% (wt/vol) yeast extract and 0.2% (wt/vol) Polypeptone (Nihon Pharmaceutical) to Sw medium. Nitrate/Yeast‐extract/Polypeptone/Seawater (NYPSw) medium was prepared by adding 10 mM sodium nitrate to the YPSw medium. Yeast‐extract/Seawater (YSw) medium was prepared by adding 0.1% (wt/vol) yeast extract to Sw medium.

The medium was dispensed by 20 ml into each 50‐ml serum bottle. Dissolved air in SPYSw, NYPSw, and YSw media was removed by flushing with N_2_:CO_2_ (4:1 [vol/vol]), and dissolved air in YPSw media was removed by flushing with H_2_:CO_2_ (4:1) at an approximate pressure of 0.15 MPa. The bottles were sealed with butyl rubber stoppers. The pH of the medium was adjusted to 7.0 with 10 mM NaHCO_3_.

*Prolixibacter bellariivorans* F2^T^ (Holmes et al., 2007) was obtained with JCM accession number of JCM 13498^T^ from the Japan Collection of Microorganisms of the RIKEN Bioresource Center (RIKEN‐BRC JCM).

### 2.2 Specimens for bacterial isolation

Crude oil emulsion samples were collected from an oil‐production well in Akita Prefecture and two crude oil storage tanks, one in Kagoshima Prefecture and the other in Miyagi Prefecture, Japan. A corrosion‐scale sample was collected from the inner surface of a cast‐iron pipe for brine transportation at a natural‐gas‐ and iodine‐production plant in Chiba Prefecture, Japan. Each of these samples was kept in a transparent oxygen‐barrier plastic bag containing an AnaeroPack‐Anaero sachet (Mitsubishi Gas Chemical) until inoculation in fresh media.

### 2.3 Enrichment, isolation, and cultivation of bacterial strains from crude oil and corrosion‐scale samples

Half a milliliter of each crude oil sample or 1.0 g of the corrosion‐scale sample was added to 20 ml of SPYSw and YPSw media, and cultivated at 25°C for 3 weeks. Each of the resultant cultures was diluted 40‐fold in the same medium and cultivated again for 3 weeks. This procedure was repeated several times. Finally, each of the enriched cultures was streaked on 1.5% (w/v) agar slants of the same medium, and cultivated anaerobically for 7 days to isolate a single colony.

### 2.4 Growth characterization

For the growth tests of each bacterial isolate, a preculture was prepared by growing an isolate in the NYPSw medium described above at 25°C for 30 days. Then, 0.1 ml of the preculture was used to inoculate in 10 ml of Sw medium supplemented with various organic acids at 10 mM. The culture was then grown at 25°C for 30 days either aerobically or anaerobically. The resulting growth was determined by measuring an optical density at 660 nm.

### 2.5 Microscopy

Routine microscopic observations were performed using an Optiphot microscope (Nikon) and an S4E stereomicroscope (Leica). The morphology of the bacterial cells was observed using a SEM (JSM‐6340F; JEOL). For scanning electron microscopy, cells were fixed in 0.1 M cacodylate buffer (pH 7.4) containing 4% (wt/vol) paraformaldehyde and 4% (wt/vol) glutaraldehyde at 4°C for 2 h, washed with 0.1 M cacodylate buffer (pH 7.4) once, fixed again in 0.1 M cacodylate buffer (pH 7.4) containing 2% (wt/vol) glutaraldehyde at 4°C for overnight, washed again in 0.1 M cacodylate buffer (pH7.4), and fixed once again in 1% (wt/vol) tannic acid in 0.1 M cacodylate buffer (pH 7.4) at 4°C for 2 h. Fixed cells were washed four times in 0.1 M cacodylate buffer (pH7.4), and post‐fixed with 2% (wt/vol) osmium tetroxide in 0.1 M cacodylate buffer (pH 7.4) at 4°C for 3 h. Samples were dehydrated in an ethanol series [50%, 70%, 90%, and 98% (vol/vol)] each for 30 min, transferred into *t*‐butyl alcohol, freeze‐dried under a vacuum, and coated with a thin layer of osmium by using an osmium plasma coater (NL‐OPC80NS; Nippon Laser and Electron Laboratory). The SEM was also used to observe the surfaces and cross‐sections of corroded Fe^0^ foils.

### 2.6 Determination of 16S rRNA gene sequences

For the phylogenetic analyses of bacterial isolates, cells grown in NYPSw medium at 25°C for 30 days were harvested, and genomic DNA was extracted from the cells by the method of Saito and Miura (1963), and quantified using Qubit dsDNA HS assay kit (Thermo Fisher Scientific). The 16S rRNA gene was amplified by PCR with primers 27F (5′‐AGAGTTTGATCCTGGCTCAG‐3′; positions 8–27 in the *Escherichia coli* numbering system) and 1492R (5′‐GGTTACCTTGTTACGACTT‐3′; positions 1510–1492). The PCR mixture (50 µl) contained 1 × PCR buffer, 2 mM MgCl_2_, 0.2 mM deoxynucleoside triphosphates (dNTPs), 1.25 U TaKaRa Ex Taq (Takara Bio Inc.) and 0.4 µM each of forward and reverse primers. Approximately 100 ng of genomic DNA was used as a template under the following cycling conditions: initial activation at 95°C for 1 min, followed by 25 cycles of denaturation at 95°C for 30 s, annealing at 50°C for 30 s, extension at 72°C for 60 s and a final extension step at 72°C for 2 min. The PCR product was purified with the QIAquick PCR purification kit (QIAGEN), and an almost‐complete 16S rRNA gene sequence (1444 bp) was determined by Sanger sequencing on a SeqStudio genetic analyzer (Applied Biosystems) with the BigDye terminator v3.1 cycle sequencing kit (Applied Biosystems) and one of the following six primers: 27F, 520F (5′‐GTGCCAGCAGCCGCGG‐3′), 920F (5′‐AAACTCAAAGGAATTGAC‐3′), 520R (5′‐ACCGCGGCTGCTGGC‐3′), 920R (5′‐GTCAATTCCTTTGAGTTT‐3′) and 1492R.

### 2.7 Phylogenetic analyses

Following a previously described method (Iino et al., 2010), the 16S RNA gene sequences of 12 phylogenetically‐related bacteria in the order *Bacteroidales* were selected. After alignment using the ARB program (Ludwig et al., 2004), the phylogenetic tree was inferred from an alignment of 1380‐bp‐long sequences of 16S rRNA genes, and constructed using the neighbor‐joining method with CLUSTAL_X 2.0.10 (Saitou & Nei, 1987; Thompson et al., 1997). The numbers at the nodes denote the bootstrap percentages derived from 1000 replications.

### 2.8 Accession numbers

*P. denitrificans* AT004 and KGS048, and *Prolixibacter* spp. NT017 and SD074 were deposited in the RIKEN‐BRC JCM under the culture collection accession numbers JCM 18695, JCM 32015, JCM 32016, and JCM 32017, respectively. The 16S rRNA gene sequences of *P*. *denitrificans* AT004, KGS048, NT017, and SD074 were deposited in the DDBJ/EMBL/GenBank nucleotide sequence database under accession numbers LC507161, LC507162, LC507163, and LC507164, respectively.

### 2.9 Fe^0^‐corrosion test

The Fe^0^‐corrosion activities of *Prolixibacter* strains were tested in a corrosion test medium consisting of Sw medium supplemented with 100 mM HEPES buffer (pH 7.0), 10 mM nitrate, and 0.05% (wt/vol) yeast extract. Fe^0^ foils (purity >99.99%, 10 × 10 × 0.1 mm) were purchased from Sigma‐Aldrich, while foils of SS400 carbon steel (10 × 10 × 2 mm), and SUS316L stainless steel (10 × 10 × 2 mm) were obtained from Nippon Steel Corp. Before use, the foil surface of SS400 carbon steel was polished with Emery polishing sandpaper (No. 400, 3M), washed extensively with distilled water, and dried by air blowing at room temperature. Each of the foils was immersed in 20‐ml of corrosion test medium contained in a 50‐ml serum bottle; the air was removed from the medium by flushing with N_2_:CO_2_ (4:1), and the bottle was sealed with a butyl rubber stopper (Nichiden‐Rika Glass). Then, the foil of Fe^0^, SS400 carbon steel, or SUS316L stainless steel were sterilized by autoclave at 121°C for 15 min. before the Fe^0^‐corrosion test. Twenty milliliters of the medium were added anaerobically and aseptically to a 50‐ml serum bottle containing a foil of Fe^0^, SS400 carbon steel, or SUS316L stainless steel. Subsequently, 0.2 ml of a bacterial preculture was added to the medium, and the culture was incubated at 25°C for 30 days.

### 2.10 Chemical analyses

After cultivation, culture fluids (100 µl) containing oxidized iron were acidified with 50 µl of 6 N HCl, and reduced with 100 µl of 1 M ascorbic acid for the quantification of total iron (ferrous and ferric ions). The iron ion concentration in each of the acidified solutions was determined colorimetrically using *o*‐phenanthroline, as described by Sandell (1959). For the quantification of nitrate, nitrite, and ammonium ions, the culture fluids were centrifuged at 20,400× *g* for 10 min. The supernatant was recovered, and filtered through a 0.2‐µm pore membrane filter. Nitrate, nitrite, and ammonium ions in the culture were quantified using a high‐performance liquid chromatography (HPLC) system (model HIC‐20Asuper; Shimadzu Corp.) equipped with a conductivity detector (model CDD‐10ADsp), a Shim‐Pack cation column (IC‐C4), and a Shim‐Pack anion column (IC‐SA2). Corrosion products on the surface of iron coupons were also analyzed using an XRD analyzer with CuKa radiation ranging from 2 *θ* = 5 to 100° at a scanning rate of 1°/min (Rint1500; Rigaku).

### 2.11 Measurement of corrosion potential and corrosion current of an Fe^0^ electrode

Electrochemical analyses were conducted at 25°C in an electrochemical cell (8 ml in capacity) equipped with three electrodes, as described by Okamoto et al. (2014), with slight modifications as follows. The working electrode was an Fe^0^ foil with a surface area of 3.14 cm^2^, which was placed on the bottom of the electrochemical cell, while the counter and reference electrodes were a platinum wire and an Ag/AgCl/(saturated KCl) electrode, respectively. A filter‐sterilized corrosion test medium was used as an electrolyte into which a cell suspension was injected to a final optical density at 660 nm of 0.02 to start the measurement. The corrosion potential of the working electrode was measured continuously, except that, every 8 h, the corrosion potential of the working electrode was swept at ±25 mV versus the corrosion potential for the measurement of the corrosion current of the working electrode.

## 3 RESULTS

### 3.1 Characterization of *Prolixibacter* strains isolated from crude oil‐ and corrosion‐scale samples

Previously, *P*. *denitrificans* MIC1‐1^T^ was isolated from a crude oil emulsion sample collected from an oil well in Akita Prefecture, Japan (Iino, Ito, et al., 2015; Iino, Sakamoto, et al., 2015). In this study, a similar method was used to isolate pure bacterial cultures from three crude oil emulsion samples and one corrosion‐scale sample collected from four different locations (Table A1). Bacteria capable of growing anaerobically in two different media, SPYSw and YPSw, were screened by repeated “dilution and growth” cycles as described in the Materials and Methods. A total of 76 pure cultures were obtained, which were characterized by 16S rRNA gene sequencing. As shown in Table A1, 16 *Prolixibacter* strains whose 16S rRNA gene sequences showed >95% identity to that of *P*. *denitrificans* MIC1‐1^T^ were isolated using SPYSw medium, while only one *Prolixibacter* strain was obtained when YPSw medium was used for the screening. In the latter medium, bacteria belonging to the genera *Aeromonas*, *Arcobacter*, *Marinilabilia*, and *Thiomicrospira* were the predominant bacteria.

**FIGURE 1:**
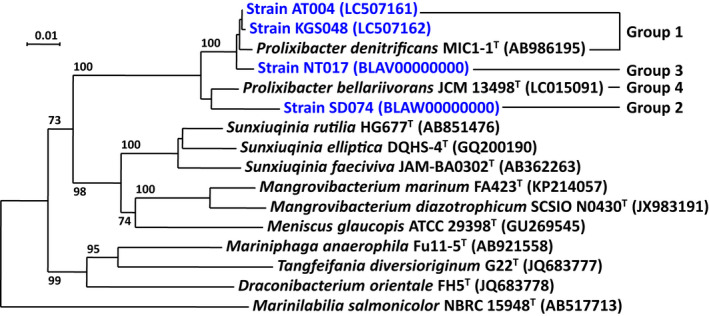
Phylogenetic tree of four bacterial isolates and representatives of related species based on the 16S rRNA gene sequences. Scale bar: 0.01 substitutions per nucleotide position

Only one *Prolixibacter* culture from a single source was used for further studies. Three pure cultures derived from three different crude oil samples were designated strains AT004, KGS048, and SD074, while one pure culture obtained from a corrosion‐scale sample was designated strain NT017 (Table 1). Phylogenetic analysis based on 16S rRNA gene sequences showed that all four isolates formed a cluster with *P*. *denitrificans* MIC1‐1^T^ and *P*. *bellariivorans* JCM 13498^T^ (original strain name is F2^T^) in the neighbor‐joining (NJ) tree, which was supported by a bootstrap value of 100%. Thus, all the isolates were accommodated in the genus *Prolixibacter*, order *Marinilabiliales*, and phylum *Bacteroidetes* (Figure 1). Among the four isolates, strains AT004, KGS048, and NT017 were closely related to *P*. *denitrificans* MIC1‐1^T^, with 98.4%–99.2% identity in their 16S rRNA gene sequences, whereas strain SD074 was phylogenetically distinct from *P*. *bellariivorans* JCM 13498^T^ and *P*. *denitrificans* MIC1‐1^T^, with pairwise sequence identities of 97.3% and 95.6%, respectively (Table A2).

**TABLE 1:** Bacterial strains used in this study

Species	Strain no.	Other designations	Sources	Reference
*Prolixibacter denitrificans*	MIC1‐1^T^	JCM 18694, DSM 27267, NBRC 102688	Crude oil from an oil well, Akita, Japan	Iino, Sakamoto, et al. (2015))
	AT004	JCM 18695	Crude oil from an oil well, Akita, Japan	This study
	KGS048	JCM 32015	Crude oil from a crude oil storage tank, Kagoshima, Japan	This study
*Prolixibacter* sp.	SD074	JCM 32017	Crude oil from a crude oil storage tank, Miyagi, Japan	This study
*Prolixibacter* sp.	NT017	JCM 32016	Corrosion scales from a cast iron pipe, Chiba, Japan	This study
*Prolixibacter bellariivorans*	F2^T^	JCM 13498, ATCC BAA‐1284	Marine‐sediment	Holmes et al. (2007)

Abbreviations: ATCC, American Type Culture Collection; DSM, German Collection of Microorganisms and Cell Cultures; JCM, Japan Collection of Microorganisms at RIKEN; NBRC, Biological Resource Center at National Institute of Technology and Evaluation.

**FIGURE A1:**
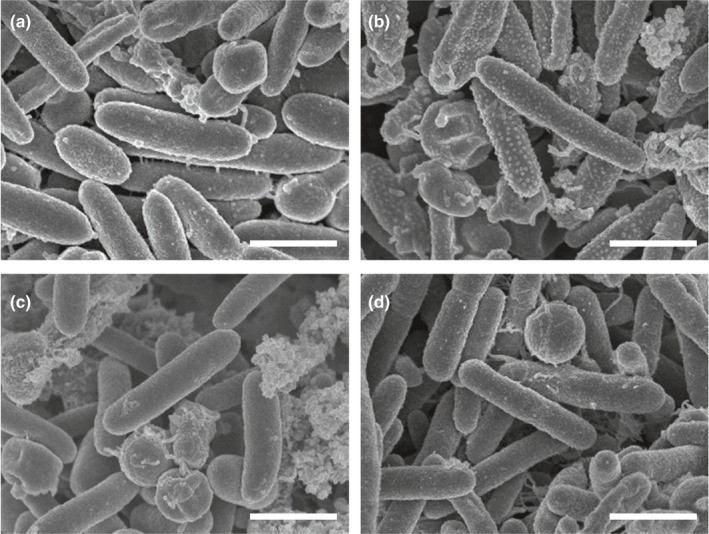
Scanning electron micrographs of cells of *P*. *denitrificans* AT004 (a), *P*. *denitrificans* KGS048 (b), *Prolixibacter* sp. NT017 (c), and *Prolixibacter* sp. SD074 (d). Scale bar: 1.0 µm

The cells of all the isolates were mainly rods with a width of approximately 0.3–0.5 µm and a length of approximately 1.2–6.5 µm and had rough cell surfaces (Figure A1). Spherical cells with a size of 0.6–0.8 µm and long rod cells with a length of 15 µm or more were observed sometimes. Cells usually occurred singly or in pairs. Motility and spore formation were not observed during phase‐contrast microscopy. The cell pellets of strains AT004 and KGS048 collected using centrifugation were salmon pink, while those of strains NT017 and SD074 were beige. The cells of strains AT004, KGS048, NT017, and SD074 were stained Gram‐negatively by conventional Gram staining (Table A2).

**FIGURE 2:**
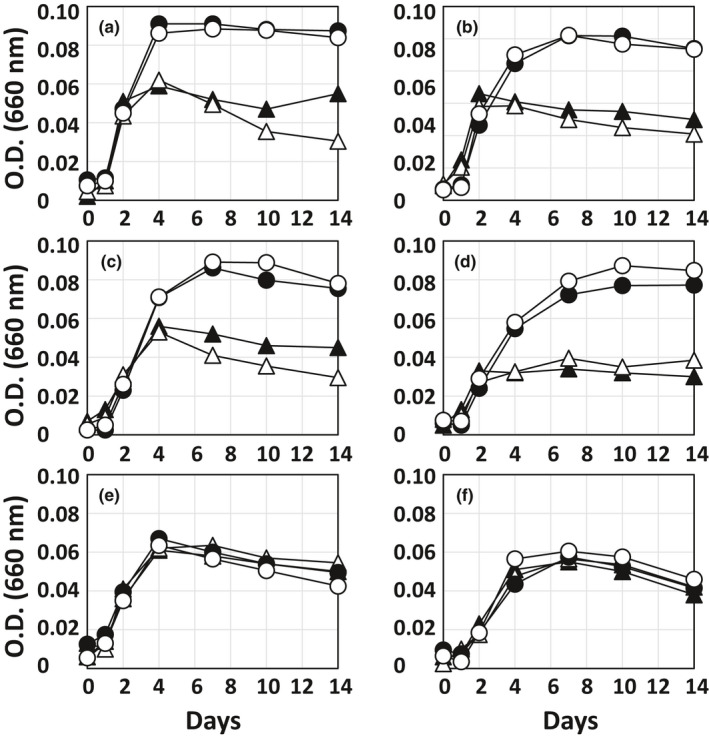
Anaerobic growth of *P*. *denitrificans* MIC1‐1^T^ (a), *P*. *denitrificans* AT004 (b), *P*. *denitrificans* KGS048 (c), *Prolixibacter* sp. SD074 (d). *Prolixibacter* sp. NT017 (e), and *P*. *bellariivorans* JCM 13498^T^ (f). These strains were grown anaerobically in either YSw medium (open symbol) or ammonium‐free YSw medium (closed symbol) in the presence (circle) or absence (triangle) of 10 mM sodium nitrate. Data represent the means (*n* = 3), with standard deviation values less than 18% of the corresponding mean values

All *Prolixibacter* strains grew anaerobically, with the same growth yields, in Sw medium supplemented with 0.1% (wt/vol) yeast extract (YSw medium) and YSw medium devoid of NH_4_Cl (ammonium‐free YSw medium) (Figure 2). Ammonium was formed upon the growth of these strains in an ammonium‐free YSm medium (Table 2), indicating that this compound was generated by the catabolism of amino acids and other nitrogen‐containing compounds present in yeast extract. Thus, yeast extract served as sources of carbon, energy, and nitrogen for the growth of the *Prolixibacter* strains in ammonium‐free YSw medium. The anaerobic growth of *P*. *denitrificans* MIC1‐1^T^ and three newly isolated strains (AT004, KGS048, and SD074) was enhanced in the presence of nitrate (Figure 2) showing that nitrate respiration improved the growth yield of these strains. On the other hand, neither growth stimulation by nitrate nor the reduction of nitrate was observed in strain NT017 and *P*. *bellariivorans* JCM 13498^T^ (Figure 2 and Table 2), indicating that these two strains did not respire nitrate. The ammonium concentrations in the nitrate‐amended cultures of the nitrate‐reducing (NR^+^) strains were significantly higher than those in the nitrate‐free cultures of the same strains (*p *< 0.05, Student's *t*‐test), while such trends were not observed in the strains unable to reduce nitrate (NR^–^) (Table 2). Thus, it seems that the NR^+^ strains reduced nitrate not only to nitrite but also to ammonium. The sum of the nitrite and ammonium concentrations formed during the cultivation of the NR^+^ strains were always smaller than the concentrations of nitrate metabolized by these strains. This stoichiometric anomaly could be interpreted as either that ammonium was assimilated by hosts, or that nitrate was also converted to other products than nitrite and ammonium, *for example*. nitric oxide.

**TABLE 2:** Nitrate reduction and/or the production of nitrite and/or ammonium during the anaerobic growth of *Prolixibacter* strains cultured in ammonium‐free YSw medium in the presence and absence of nitrate and ammonium.

Species	Strain	NO_3_	NH_4_	Nitrate reduced (mM)	Nitrite formed (mM)	Ammonium formed (mM)
*Prolixibacter denitrificans*	MIC1‐1^T^	–	–	N.A.	N.A.	1.1 ± 0.11
		–	+	N.A.	N.A.	1.0 ± 0.27
		+	–	5.1 ± 0.94	1.8 ± 0.65	1.4 ± 0.41
		+	+	4.8 ± 0.97	3.3 ± 1.14	1.4 ± 0.19
*Prolixibacter denitrificans*	AT004	–	–	N.A.	N.A.	0.5 ± 0.07
		–	+	N.A.	N.A.	0.5 ± 0.03
		+	–	5.2 ± 0.07	2.5 ± 0.07	1.0 ± 0.39
		+	+	5.0 ± 0.07	2.6 ± 0.07	1.1 ± 0.14
*Prolixibacter denitrificans*	KGS048	–	–	N.A.	N.A.	0.6 ± 0.11
		–	+	N.A.	N.A.	0.6 ± 0.08
		+	–	4.8 ± 0.07	1.7 ± 0.07	0.9 ± 0.27
		+	+	5.0 ± 0.07	3.2 ± 0.07	1.0 ± 0.35
*Prolixibacter* sp.	SD074	–	–	N.A.	N.A.	0.3 ± 0.05
		–	+	N.A.	N.A.	1.0 ± 0.58
		+	–	5.6 ± 1.19	2.0 ± 0.67	1.2 ± 0.16
		+	+	6.8 ± 0.65	4.4 ± 0.50	1.6 ± 0.05
*Prolixibacter* sp.	NT017	–	–	N.A.	N.A.	0.8 ± 0.08
		–	+	N.A.	N.A.	0.8 ± 0.04
		+	–	0.4 ± 0.29	<0.01	0.4 ± 0.08
		+	+	0.3 ± 0.16	<0.01	0.6 ± 0.16
*Prolixibacter bellariivorans*	JCM 13498^T^	–	–	N.A.	N.A.	0.6 ± 0.06
		–	+	N.A.	N.A.	0.6 ± 0.07
		+	–	0.4 ± 0.29	< 0.01	0.4 ± 0.14
		+	+	0.2 ± 0.25	< 0.01	0.6 ± 0.12

Note

Each of the six *Prolixibacter* strains was grown anaerobically at 25°C for 30 days under an atmosphere of N_2_:CO_2_ (4:1) in a 50‐ml serum bottle containing 20 ml of ammonium‐free YSw medium or the medium supplemented with 10 mM nitrate and/or 2.8 mM ammonium. The concentrations of nitrate, nitrite, and ammonium in each culture or in an aseptic control at day 30 were determined, and those in the aseptic control were subtracted from the respective values in each culture. Data represent means and standard deviations (*n* = 3).

Abbreviation: N.A., not applicable.

Yeast extract in YSm medium could be replaced by d‐glucose as sole carbon and energy sources, but not by simple organic acids, including lactate, pyruvate, and acetate (Table A2).

Based on the phylogenetic positions shown in Figure 1, the phenotypic properties shown in Table A2, and the nitrate‐reducing activities described above, strains AT004 and KGS048 are considered to belong to *P*. *denitrificans*. Strain NT017, whose 16S rRNA gene sequence was 98.9% identical to that of *P*. *denitrificans* MIC1‐1^T^, differed from *P*. *denitrificans* by its cell color and the absence of nitrate respiration. Strain SD074 was considered to be a new species because of the low identity of its 16S rRNA gene sequence compared with those of *P*. *bellariivorans* and *P*. *denitrificans*.

### 3.2 Corrosion of Fe^0^ by *Prolixibacter* strains

When *P*. *denitrificans* MIC1‐1^T^ and three newly isolated strains (*P*. *denitrificans* AT004, *P*. *denitrificans* KGS048, and *Prolixibacter* sp. SD074) were grown in a corrosion test medium containing Fe^0^ foils, the surface of the Fe^0^ foil lost its shine and turned grayish‐black within one month of their cultivation. In addition, the color of the medium changed to light yellow, suggestive of the formation of ferric ions in the cultures. On the other hand, these color changes were not observed in the anaerobic cultures of *Prolixibacter* sp. NT017 or *P*. *bellariivorans* JCM 13498^T^. Thus, all nitrate reducers were expected to be Fe^0^‐corroding, while all nitrate non‐reducers were not.

**FIGURE 3:**
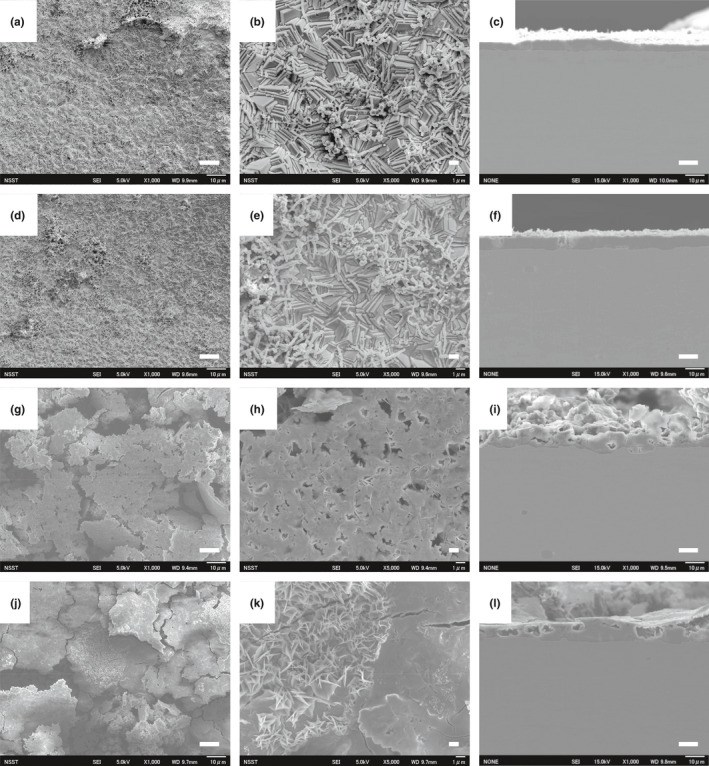
Scanning electron micrographs showing the surface and cross‐sections of Fe^0^ foils incubated anaerobically with NR^+^ *Prolixibacter* strains. Fe^0^ foils were incubated for 30 days in a corrosion test medium in the presence of *P*. *denitrificans* MIC1‐1^T^ (a–c), *P*. *denitrificans* AT004 (d–f), *P*. *denitrificans* KG048 (g–i), and *Prolixibacter* sp. SD074 (j–l). a, d, g, and j: The surface of Fe^0^ foils (×300 magnification). b, e, h, and k: The surface of Fe^0^ foils (×1000 magnification). c, f, i, and l: The cross‐section of Fe^0^ foils (×300 magnification). Scale bar: 10 µm for a, c, d, f, g, i, j, and l; 1 µm for b, e, h, and k. Scanning electron micrographs were obtained from two independent experiments

**FIGURE 4:**
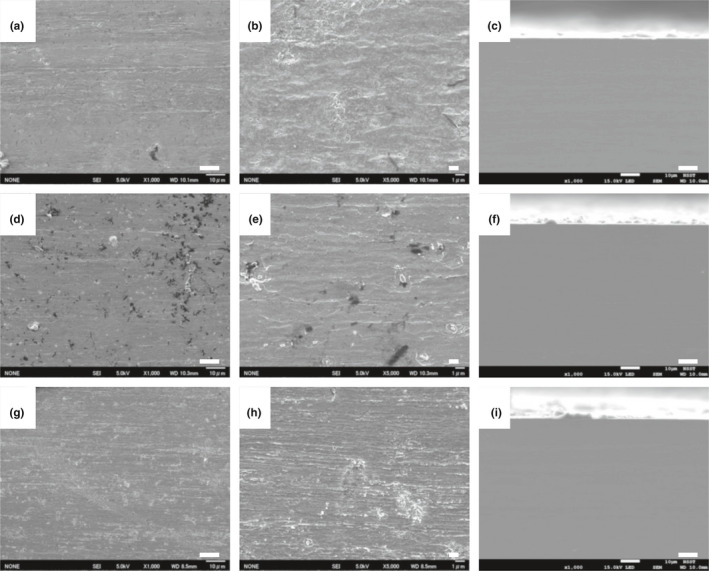
Scanning electron micrographs showing the surface and cross‐sections of Fe^0^ foils incubated anaerobically with NR^–^ *Prolixibacter* strains. Fe^0^ foils were incubated for 30 days in the corrosion test medium in the presence of *Prolixibacter* sp. NT017 (a–c), and *P*. *belleriivorans* JCM 13498^T^ (d–f). The aseptic controls are also shown (g–i). a, d, and g: the surface of Fe^0^ foils (×300 magnification). b, e, and h: the surface of Fe^0^ foils (×1000 magnification). c, f, and i: the cross‐section of Fe^0^ foils (×300 magnification). Bar = 10 µm for a, c, d, f, g, and i. Scale bar: 1 µm for b, e, and h. Scanning electron micrographs were obtained from two independent experiments

To investigate further, Fe^0^ foils incubated in the corrosion test medium for 30 days in the presence or absence of various *Prolixibacter* strains were analyzed with a scanning electron microscope (SEM). As shown in Figure 3a,b,d,e, cubic crystals developed on the surface of Fe^0^ foils after incubation with *P*. *denitrificans* MIC1‐1^T^ and *P*. *denitrificans* AT004. Rod‐shaped cells of length 1.5–6 µm were also observed on the surface of Fe^0^ foils (Figure 3b,e). The cross‐sections of Fe^0^ foils showed that the surface was eroded after incubation with these strains (Figure 3c,f) compared with the aseptic control (Figure 4i). On the surfaces of Fe^0^ foils submerged in the cultures of *P*. *denitrificans* KGS048 and *Prolixibacter* sp. SD074, amorphous flakes developed (Figure 3g–l), and the cross‐sectional morphology of the corrosion products showed a greater thickness of the corrosion films (Figure 3i,l) than those produced by *P*. *denitrificans* MIC1‐1^T^ and AT004 (Figure 3c,f). On the other hand, the amounts of corrosion deposits on the surface of the Fe^0^ foils submerged in the cultures of nitrate non‐reducers, namely *Prolixibacter* sp. NT017 and *P*. *bellariivorans* JCM 13498^T^, were minimal (Figure 4a–f), as well as similar to those in the aseptic control (Figure 4g–i).

The X‐ray diffraction analyses of the corroded Fe^0^ samples revealed that deposits developed on the surface of Fe^0^ foil from the cultures of *P*. *denitrificans* (MIC1‐1^T^, AT004, and KGS048) and *Prolixibacter* sp. SD074 mainly consisted of FeCO_3_ and Fe_3_(PO_4_)_2_ (Table A3). In one of the two samples from the cultures of *P*. *denitrificans* KGS048 and *Prolixibacter* sp. SD074, Fe_3_O_4_Fe_2_O_3,_ and FeO(OH) were also detected on the surface of Fe^0^ foils. A very thin layer of Fe_3_(PO_4_)_2_ was detected on the surface of Fe^0^ foils in the cultures of *P*. *bellariivorans* JCM 13498^T^ and the aseptic control, while corrosion products were not detected on the surface of the Fe^0^ foils in the culture of *Prolixibacter* sp. NT017. Thus, the electron microscopic studies confirmed the conclusions obtained by visual observation, that is, that the four NR^+^ strains *P*. *denitrificans* MIC1‐1^T^, *P*. *denitrificans* AT004, *P*. *denitrificans* KGS048, and *Prolixibacter* sp. SD074 were Fe^0^‐corrosive, while the two NR^–^ strains, *P*. *bellariivorans* JCM 13498^T^, and *Prolixibacter* sp. NT017 were not.

**FIGURE 5:**
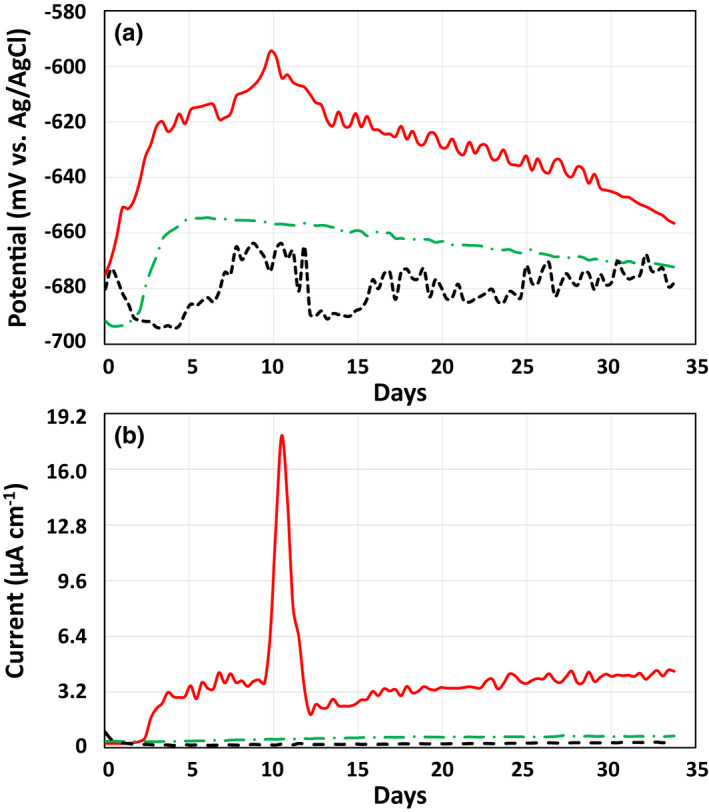
Corrosion potential and corrosion current of an Fe^0^ electrode immersed in the cultures of *P*. *denitrificans* strain MIC1‐1^T^ and *Prolixibacter* sp. NT017. Corrosion potential and corrosion current of an Fe^0^ electrode were determined as described in the Materials and Methods. (a) Change in the corrosion potential of an Fe^0^ foil used as the working electrode. (b) Change in the corrosion current generated at the Fe^0^ foil poised at 25 mV versus corrosion potential. Solid red line: in the presence of *P*. *denitrificans* MIC1‐1^T^; dashed green line: in the presence of *Prolixibacter* sp. strain NT017; dotted black line, aseptic control

Changes in the corrosion potential of Fe^0^ (= working electrode) immersed in the corrosion test medium in the presence or absence of a *Prolixibacter* strain were examined, as shown in Figure 5a. In the presence of Fe^0^‐corroding *P*. *denitrificans* MIC1‐1^T^, the corrosion potential shifted in the positive direction from −680 mV (vs. KCl saturated Ag/AgCl) to −600 mV in the first 10 days, followed by a shift in the negative direction. Such intensive changes in the corrosion potential were not observed in the aseptic control or in the presence of Fe^0^‐non‐corroding *Prolixibacter* sp. NT017. Every 8 h during the continuous measurement of the corrosion potential of the working electrode, the corrosion current was also estimated by sweeping ±25 mV around the corrosion potential. As shown in Figure 5b, the current density increased between days 2 and 4 from almost zero to 3.2 µA/cm. After day 4, the current density remained almost unchanged, except between days 10 and 12, in which it increased temporarily up to approximately 19 µA/cm (Figure 5b). On the other hand, no corrosion current (<0.5 µA/cm) was generated upon the polarization of Fe^0^ in the aseptic control and in the presence of *Prolixibacter* sp. NT017.

### 3.3 Fate of nitrate during Fe^0^ corrosion

As has been observed in our previous study (Iino, Ito, et al., 2015), *P*. *denitrificans* MIC1‐1^T^ grown in corrosion test medium containing 10 mM nitrate corroded Fe^0^ to extents more than 20‐fold higher than that in the aseptic control. When yeast extract in corrosion test medium was substituted by _D_‐glucose, Fe^0^ oxidation by *P*. *denitrificans* MIC1‐1^T^ was reduced by 50% probably due to the inhibitory effect of _D_‐glucose on Fe^0^ corrosion (Isa et al., 2012). The three newly isolated NR^+^ strains, *P*. *denitrificans* AT004, *P*. *denitrificans* KGS048, and *Prolixibacter* sp. SD074, also oxidized Fe^0^ extensively (Table 3). Conversely, the two NR^–^ strains, *Prolixibacter* sp. NT017 and *P*. *bellariivorans* JCM 13498^T^ did not show such an activity confirming the results from the electron microscopic and electrochemical studies. None of the six strains enhanced Fe^0^ corrosion in the presence of sulfate in place of nitrate (Table 3).

**TABLE 3:** Fe^0^ corrosion and nitrate reduction to nitrite and ammonium in the cultures of *Prolixibacter* strains

Species	Strain	Electron acceptor	Carbon source	Oxidized iron (mM)			Nitrate reduced (mM)	Nitrite formed (mM)	Ammonium formed (mM)
				Total iron (mM)	Fe^2+^ (mM)	Fe^3+^ (mM)			
*Prolixibacter denitrificans*	MIC1‐1^T^	Nitrate	Glucose	2.9 ± 0.01	2.8 ± 0.02	0.1 ± 0.02	3.3 ± 1.3	0.1 ± 0.01	0.5 ± 0.9
		Nitrate	Yeast ex.	4.7 ± 0.5	2.2 ± 0.2	2.1 ± 0.5	5.1 ± 0.3	0.4 ± 0.07	2.7 ± 0.1
		Sulfate	Yeast ex.	0.1 ± 0.02	–	–	–	–	–
*Prolixibacter denitrificans*	AT004	Nitrate	Yeast ex.	4.3 ± 0.3	3.0 ± 0.1	1.5 ± 0	5.3 ± 0.3	0.3 ± 0.04	1.7 ± 0.1
		Sulfate	Yeast ex.	0.5 ± 0.09	–	–	–	–	–
*Prolixibacter denitrificans*	KGS048	Nitrate	Yeast ex.	4.8 ± 0.4	2.8 ± 0.5	1.3 ± 0.2	5.4 ± 0.4	0.3 ± 0.08	1.8 ± 0.1
		Sulfate	Yeast ex.	0.2 ± 0.11	–	–	–	–	–
*Prolixibacter* sp.	SD074	Nitrate	Yeast ex.	5.4 ± 0.5	2.8 ± 0.4	2.4 ± 0.4	4.9 ± 0.2	0.6 ± 0.04	2.3 ± 0.1
		Sulfate	Yeast ex.	0.3 ± 0.01	–	–	–	–	–
*Prolixibacter* sp.	NT017	Nitrate	Yeast ex.	0.2 ± 0.03	0.2 ± 0.01	0 ± 0.01	0 ± 0.13	< 0.01	1.1 ± 0.08
		Sulfate	Yeast ex.	0.3 ± 0.07	–	–	–	–	–
*Prolixibacter bellariivorans*	JCM 13498^T^	Nitrate	Yeast ex.	0.2 ± 0.10	0.2 ± 0.01	0 ± 0.01	0 ± 0.13	< 0.01	0.9 ± 0.08
		Sulfate	Yeast ex.	0.2 ± 0.20	–	–	–	–	–

Note

Each of the six *Prolixibacter* strains was grown anaerobically at 25°C for 30 days under an atmosphere of N_2_:CO_2_ (4:1) in a 50‐ml serum bottle containing an Fe^0^ foil immersed in 20 ml of corrosion test medium or the medium containing 10 mM sulfate in place of nitrate. The concentrations of oxidized iron, nitrate, nitrite, and ammonium in each culture at day 30 were determined, and those in the aseptic control were subtracted from the respective values in each culture. Data represent means and standard deviations (*n* = 3). –: Not determined.

In Fe^0^‐foil‐containing corrosion test media inoculated with the NR^+^ strains, nitrate was reduced by 50% over 30 days (Table 3), similarly to the results in Table 2. On the other hand, the nitrite concentrations were lower, while the ammonium concentrations were higher in the Fe^0^‐containing cultures (Table 3) compared to Fe^0^‐free cultures (Table 2). From these results, and from the previous finding that nitrite chemically corrodes Fe^0^ (Alowitz & Scherer, 2002), we hypothesized that Fe^0^ was chemically oxidized to Fe^2+^ and/or Fe^3+^ concomitantly with the reduction of nitrite to ammonium.

**FIGURE 6:**
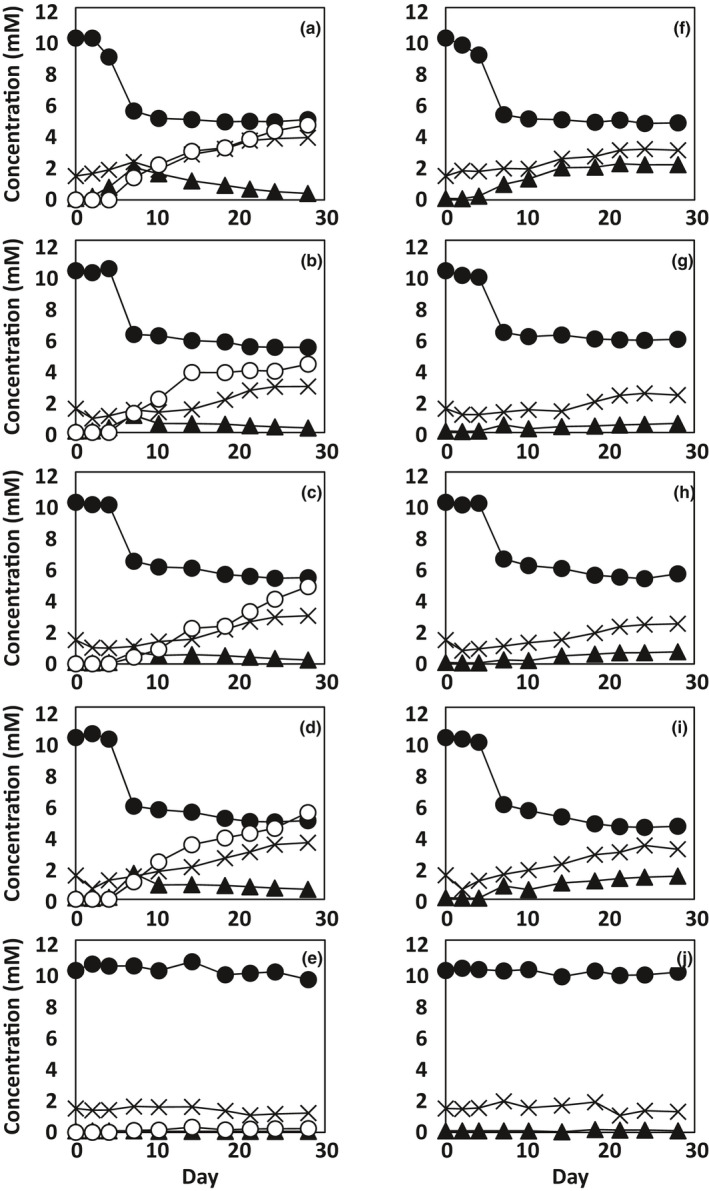
Nitrate reduction and the accumulation of nitrite and ammonium in the cultures of NR^+^ *Prolixibacter* strains. NR^+^ *Prolixibacter* strains were grown under an atmosphere of N_2_:CO_2_ (4:1) in corrosion test medium either in the presence (a to e) or absence (f to j) of an Fe^0^ foil. In f to j, the concentrations of oxidized iron in the aseptic controls were between 0 mM at day 0 and 0.2 mM at day 28. The strains used were *P*. *denitrificans* MIC1‐1^T^ (A and F), *P*. *denitrificans* AT004 (b and g), *P*. *denitrificans* KGS048 (C and H) *Prolixibacter* sp. SD074 (d and i), and aseptic control (e and j). Filled circle: nitrate concentration, filled triangle: nitrite concentration, cross: ammonium concentration, and open circles: concentration of oxidized iron. Data represent means (*n* = 3), with standard deviation values less than 8.9% of the corresponding mean values

To clarify this point, time‐course changes in the concentrations of nitrate, nitrite, and ammonium in the cultures of the four NR^+^ strains in the presence or absence of Fe^0^ were examined for four weeks. As shown in Figure 6, a similar trend was observed among the four strains for the concentration changes of nitrate, nitrite, and ammonium. In all the cultures (Figure 6a–d,f–i), the nitrate concentrations decreased sharply during the first week, followed by gradual decreases.

In the Fe^0^‐non‐amended cultures (Figure 6a–d), the ammonium concentrations decreased during the first 2 to 4 days, indicating that a portion of ammonium present in the corrosion test medium was used as a nitrogen source during the growth of these strains. Subsequently, the ammonium concentrations increased. The nitrite concentrations in the same cultures increased continuously until the end of the cultivation period.

**FIGURE 7:**
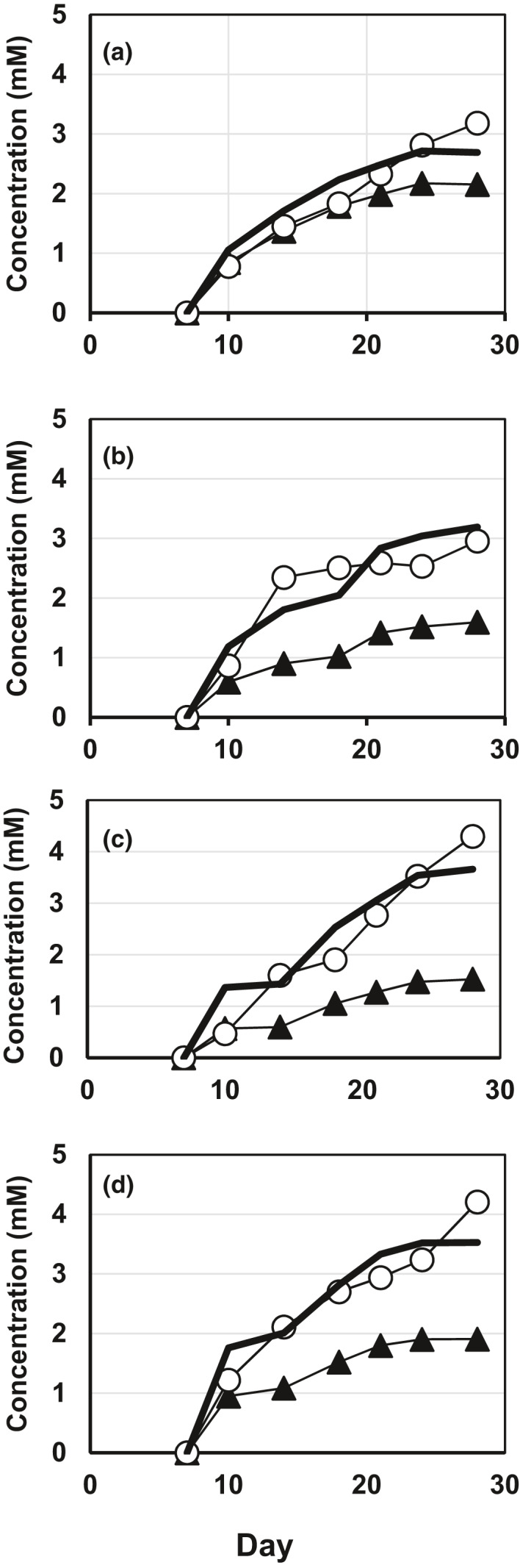
The relationship between Fe^0^ oxidation and nitrite reduction. The data in Figure 6 are rearranged to show the relationships between the increment in the concentration of oxidized iron (open circles) and the consumed concentration of nitrite (filled triangle) during three weeks after day 7. The latter concentration was calculated as described in the text. The strains used were *P*. *denitrificans* MIC1‐1^T^ (a), *P*. *denitrificans* AT004 (b), *P*. *denitrificans* KGS048 (c), and *Prolixibacter* sp. SD074 (d). Bold lines were obtained by multiplying the values of the consumed concentration of nitrite by *a* where a = 1.25 for a, 2 for b, 2.4 for c, and 1.85 for d. Data represent means (*n* = 3), with standard deviation values less than 8.9% of the corresponding mean values

In the Fe^0^‐amended cultures (Figure 6f–i), the changes in the nitrite and ammonium concentrations were different compared with those in the Fe^0^‐non‐amended cultures. Compared to the ammonium concentrations in the Fe^0^‐non‐amended cultures, those in the Fe^0^‐amended cultures were similar for the first two weeks, but higher in the last two weeks. On the other hand, the nitrite concentrations in the Fe^0^‐amended cultures were highest on day 7, after which they declined. This decline was interpreted to be due to the chemical reduction of nitrite to ammonium coupled with the chemical oxidation of Fe^0^. In such a reaction, the reduction of one mole of nitrite to ammonium is coupled to the oxidation of either 2 mol of Fe^0^ to Fe^3+^ or 3 mol of Fe^0^ to Fe^2+^. To determine whether this stoichiometry was established, the relationship between the formation of oxidized iron and the consumption of nitrite was examined. As shown in Figure 7, the increment in the concentrations of oxidized iron between day 7 and day *X* (Δ[Fe*^b^* ^+^]_Day7_to__ *_X_*) was calculated accordingly using the following equation:

$$\Delta {\left[{\text{Fe}}^{b+}\right]}_{\text{Day}7\_\text{to}\_X}={\left[{\text{Fe}}^{b+}\right]}_{\text{Day}X}‐{\left[{\text{Fe}}^{b+}\right]}_{\text{Day}7}$$

where [Fe*^b^* ^+^]_Day_ *_X_* is the sum of the concentrations of Fe^2+^ and Fe^3+^ on day *X* (*X* = 10, 14, 18, 21, 24 and 28). Because the changes in the nitrite concentrations between day 7 and day *X* can be expressed as:

$${\left[{\text{NO}}_{2}^{‐}\right]}_{\text{Day}X}‐{\left[{\text{NO}}_{2}^{‐}\right]}_{\text{Day}7}=\text{syn}{\left[{\text{NO}}_{2}^{‐}\right]}_{\text{Day}7\_\text{to}\_X}‐\deg{\left[{\text{NO}}_{2}^{‐}\right]}_{\text{Day}7\_\text{to}\_X}$$

where syn[NO_2_^–^]_Day7_to__ *_X_* and deg[NO_2_^–^]_Day7_to__ *_X_* are the nitrite concentrations synthesized and degraded between day 7 and day *X*, respectively. Since syn[NO_2_^–^]_Day7_to__ *_X_* can be estimated from the decrease in the nitrate concentration during this period:

$$\text{syn}{\left[{\text{NO}}_{2}^{‐}\right]}_{\text{Day}7\_\text{to}\_X}={\left[{\text{NO}}_{3}^{‐}\right]}_{\text{Day}7}‐{\left[{\text{NO}}_{3}^{‐}\right]}_{\text{Day}x}$$

the nitrite consumption between day 7 and day *X* can be expressed as:

$$\deg{\left[{\text{NO}}_{2}^{‐}\right]}_{\text{Day}7\_\text{to}\_X}{\left[{\text{NO}}_{3}^{‐}\right]}_{\text{Day}7}‐{\left[{\text{NO}}_{3}^{‐}\right]}_{\text{Day}X}+{\left[{\text{NO}}_{2}^{‐}\right]}_{\text{Day}7}‐{\left[{\text{NO}}_{2}^{‐}\right]}_{\text{Day}X}$$

In Figure 7, the bold lines show the fitting of the function *a* × deg[NO_2_^–^]_Day7_to__ *_X_* to the Δ[Fe*^b^* ^+^]_Day7_to__ *_X_* data points, where *a* is a fitting parameter. The best fittings were obtained with *a* = 1.25, 2, 2.3, and 1.85 for Figure 7a–d, respectively. Thus, the *a* values were often smaller than the expected values (between 2 and 3), probably because (i) the extraction efficiency of oxidized iron species from Fe^0^ foils may not be 100%, and/or (ii) nitrite was reduced not only chemically by Fe^0^ and Fe^2+^ but also biologically to either ammonium or nitric oxide. In any case, the results in Figure 7 indicate that sufficient amounts of nitrite were consumed to account for the observed amounts of oxidized Fe^0^.

The chemical corrosion of Fe^0^ by nitrite under the current experimental setup was also examined. As shown in Table A4, nitrite oxidized Fe^0^ in a concentration‐dependent manner.

### 3.4 Corrosion of SS400 carbon steel and SUS316L stainless steel by *Prolixibacter* strains

Two NR^+^ strains, *P*. *denitrificans* MIC1‐1^T^ and *Prolixibacter* sp. SD074, and one NR^–^ strain, *Prolixibacter* sp. NT017, were used to evaluate the corroding activities on SS400 carbon steel and SUS316L stainless steel (Table A5) under anaerobic conditions. Both NR^+^ strains corroded SS400 carbon steel more intensively than Fe^0^ (Table 4). However, these two strains did not corrode SUS316L stainless steel, indicating that they could not accept electrons through the passive film formed on the surface of stainless steel. As expected, the Fe^0^‐non‐corroding *Prolixibacter* sp. NT017 corroded neither SS400 carbon steel nor SUS316L stainless steel.

**TABLE 4:** Corrosion activities of two Fe^0^‐corroding *Prolixibacter* strains on Fe^0^, carbon steel, and stainless steel

Species	Strain	Metal type	Oxidized iron (mM)
*Prolixibacter denitrificans*	MIC1‐1^T^	Fe^0^	4.2 ± 0.2
		SS400	9.0 ± 0.4
		SUS316L	0.1 ± 0.002
*Prolixibacter* sp.	SD074	Fe^0^	5.1 ± 0.4
		SS400	6.7 ± 0.2
		SUS316L	0.1 ± 0.002
*Prolixibacter* sp.	NT017	Fe^0^	0.2 ± 0.03
		SS400	0 ± 0.002
		SUS316L	0 ± 0.002
Aseptic control		Fe^0^	0.2 ± 0.12
		SS400	0 ± 0.002
		SUS316L	0 ± 0.002

Note

*P*. *denitrificans* MIC1‐1^T^ and *Prolixibacter* sp. SD074 grew anaerobically at 25°C for 30 days under an atmosphere of N_2_:CO_2_ (4:1) in a 50‐ml serum bottle containing either an Fe^0^ foil, a coupon of SS400 carbon steel, or a coupon of SUS316L stainless steel immersed in 20 ml of corrosion test medium. The concentrations of oxidized iron in each culture at day 30 were determined. Data represent means and standard deviations (*n* = 3).

## 4 DISCUSSION

In this study, four bacterial strains closely related to *P*. *denitrificans* MIC1‐1^T^ and *P*. *bellariivorans* JCM 13498^T^ were newly isolated from crude oil‐ or corrosion‐scale samples (Tables A1 and 1). Phylogenetically, these four strains formed a monophyletic lineage together with *P*. *bellariivorans* JCM 13498^T^ and *P*. *denitrificans* MIC1‐1^T^ with a bootstrap value of 100% (Figure 1). The pairwise 16S rRNA sequence similarities among these four strains and two previously isolated strains, *P*. *denitrificans* MIC1‐1^T^ and *P*. *bellariivorans* JCM 13498^T^, were >95.8%, which was higher than the cutoff value for the delimitation of prokaryotic genera (95%) (Stackebrandt & Goebel, 1994; Tindall et al., 2010). Thus, the four newly isolated strains belonged to the genus *Prolixibacter*. Among them, strains AT004 and KGS048 were considered to belong to *P*. *denitrificans* based on their 16S rRNA gene sequences and phenotypic traits.

Nitrate‐reducing activity was observed in some but not all strains in the genus *Prolixibacter*. The three strains belonging to *P*. *denitrificans*, namely strains MIC1‐1^T^, AT004, and KGS048, were all nitrate reducers, while *Prolixibacter* sp. NT017, a close relative of *P*. *denitrificans*, was nitrate non‐reducer (Table 2). Although *P*. *bellariivorans* JCM 13498^T^ and *Prolixibacter* sp. SD074 formed the second clade in the genus *Prolixibacter* (Figure 1), the former was nitrate non‐reducer and the latter was nitrate‐reducer. Thus, there was no concordance between the phylogenetic relationships and the nitrate‐reducing phenotype. The anaerobic growth of the NR^+^ strains was promoted by nitrate, while that of the NR^–^ strains was not (Figure 2). The growth improvement with nitrate may be due to nitrate respiration, which provides more ATP than the fermentation, and due to nitrate assimilation, which provides more organic nitrogen to the hosts. The nitrogen assimilation in the NR^+^ strains was also supported by nitrogen stoichiometry analysis in Figure 6.

Nitrate‐reducing *Prolixibacter* strains enhanced Fe^0^ corrosion, which has been demonstrated by electron microscopic studies (Figures 3 and 4), electrochemical studies (Figure 5), and the biochemical analysis of corrosion products (Table 3, Figure 6). On the other hand, the enhancement of Fe^0^‐corroding activity was not observed in the NR^–^ *Prolixibacter* strains.

**FIGURE A2:**
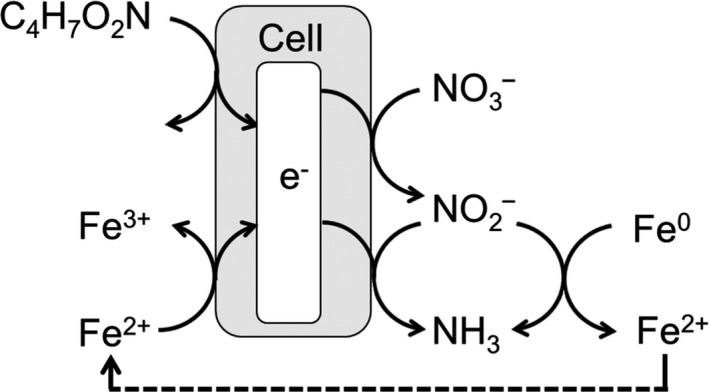
Proposed mechanism of anaerobic Fe^0^ corrosion induced by nitrate‐reducing *Prolixibacter* strains in the corrosion test medium. Electrons generated by the metabolism of yeast extract (C_4_H_7_O_2_N) are used to reduce nitrate to nitrite by nitrate reductase. Nitrite is further reduced to ammonium either biologically or chemically by the reaction coupled to the oxidation of Fe^0^ to Fe^2+^. A part of Fe^2+^ is oxidized to Fe^3+^ mainly by biological action

MIC can be classified into two types, namely chemical MIC (CMIC) and electrical MIC (EMIC), according to Fe^0^‐corrosion mechanisms (Enning et al., 2012). We propose that the NR^+^ *Prolixibacter* strains enhanced Fe^0^ corrosion mainly through CMIC, as shown in Figure A2, based on the results obtained between days 7 and 28, during which over 70% of the corrosion products were produced. One evidence that nitrite formed by biological nitrate reduction was the major causal agent of Fe^0^ corrosion came from the results presented in Figure 6, which showed the reduction of nitrite in parallel to the Fe^0^ oxidation. The amount of nitrite consumed between days 7 and 28 was sufficient to produce the observed amounts of oxidized Fe^0^ (Figure 7). This was further demonstrated by nitrite‐induced chemical Fe^0^ corrosion, as shown in Table A4. There was no evidence for biotic oxidation of Fe^0^ or Fe^2+^ coupled to the reduction of nitrate because the nitrate reduction by the NR^+^ strains was not enhanced by the presence of Fe^0^ and Fe^2+^ (Figure 6). On the other hand, the NR^+^ strains may catalyze the oxidation of Fe^2+^ coupled to the reduction of nitrite which was observed previously (Schaedler et al., 2018), because the oxidation of Fe^0^ by the NR^+^ strains formed both Fe^2+^ and Fe^3+^ in the average ratio of 3:2 (Table 3), while only a scarce amount of Fe^3+^ was formed in the abiotic oxidation of Fe^0^ (Table A4).

*P. denitrificans* MIC1‐1^T^ and *Prolixibacter* sp. SD074 induced the corrosion of SS400 carbon steel in the presence of nitrate. The corrosion rates by these strains calculated by millimeters per year were 0.15 mm/year, while the rate in the aseptic control was below 1.0×10^−3^ mm/year (Text A1). All of the iron‐corrosive *Prolixibacter* strains were isolated from either a crude oil well or crude oil storage tanks. Nitrate is widely used to prevent MIC because it enhances the growth of NRB, which competitively inhibit the growth of Fe^0^‐corroding sulfate‐reducing bacteria (Gittel et al., 2009; Schwermer et al., 2008; Telang et al., 1997). However, sulfide‐oxidizing, nitrate‐reducing bacteria such as *Sulfurimonas* sp. strain CVO (Lahme et al., 2019) have been suggested to increase corrosion after nitrate injection. This study demonstrated that NR^+^ *Prolixibacter* may be prevalent in oil fields, and thus has the potential to enhance steel corrosion in oil fields subjected to nitrate injection. Thus, understanding the distribution and abundance of NR^+^ *Prolixibacter* in oil and gas fields is important for evaluating corrosion risk associated with nitrate injection to treat reservoir souring. We are currently developing a PCR‐based method to detect environmental NR^+^ *Prolixibacter*.

## CONFLICT OF INTEREST

None declared.

## AUTHOR CONTRIBUTIONS

**Takao Iino:** Conceptualization (lead); Formal analysis (lead); Funding acquisition (lead); Investigation (lead); Writing‐original draft (lead); Writing‐review & editing (lead). **Nobuaki Shono:** Formal analysis (lead); Writing‐review & editing (equal). **Kimio Ito:** Formal analysis (equal); Writing‐review & editing (equal). **Ryuhei Nakamura:** Formal analysis (equal); Writing‐review & editing (equal). **Kazuo Sueoka:** Writing‐review & editing (equal). **Shigeaki Harayama:** Conceptualization (equal); Supervision (lead); Writing‐original draft (equal); Writing‐review & editing (equal). **Moriya Ohkuma:** Funding acquisition (equal); Supervision (equal); Writing‐original draft (equal).

## ETHICS STATEMENT

None required.

## Data Availability

All data are provided in full in this paper apart from the 16S rRNA gene sequences encompassing *P*. *denitrificans* AT004, and KGS048, *Prolixibacter* spp. NT017, and SD074, which are available in the NCBI GenBank under the accession numbers LC507161–LC507164: https://www.ncbi.nlm.nih.gov/nuccore/LC507161,LC507162,LC507163,LC507164.

## APPENDIX 1

### Text A1. Estimation of corrosion rates in mm per year

The amount of dissolved Fe^0^ from one side of a SS400 foil (10 × 10 × 2 mm) immersed in 20‐ml cultures of the nitrate‐reducing *Prolixibacter* strains was 9.0 mM per month. Since the molecular weight of Fe^0^ is 55.8, 9.0 × 55.8 × 0.02 mg (≈10.0 mg) of Fe^0^ were dissolved per month. The specific gravity of Fe^0^ is 7.85 g/cm^3^. Thus, 1.3 × 10^–3^ cm^3^ of Fe^0^ per month, or 15 × 10^−3^ cm^3^ of Fe^0^ per year were dissolved from the SS400 foil with a surface area of 1 cm^2^. These values corresponded to a corrosion rate of 0.15 mm/year.

## APPENDIX 2

**TABLE A1:** Screening of *Prolixibacter* strains from pure cultures obtained from crude oil‐ and corrosion‐scale samples

Source of isolation	Medium for enrichment^a^	Number of *Prolixibacter* isolates	Total number of isolates	Frequency (%) of *Prolixibacter* strains
Crude oil	SPYSw	4	10	40
(Akita)	YPSw	0	11	0
Crude oil	SPYSw	1	18	6
(Kagoshima)	YPSw	0	9	0
Crude oil	SPYSw	9	13	69
(Miyagi)	YPSw	0	6	0
Corrosion scales	SPYSw	2	2	100
(Chiba)	YPSw	1	7	14

^a^ SPYSw medium was prepared by adding 10 mM sulfate, 10 mM sodium pyruvate, and 0.01% (wt/vol) yeast extract to Sw medium. Cultures were grown under N_2_:CO_2_ (4:1). YPSw medium was prepared by adding 0.2% (wt/vol) yeast extract and 0.2% (wt/vol) Polypepton (Nihon Pharmaceutical) to Sw medium. Cultures were grown under H_2_–CO_2_ (4:1 at approximately 150 kPa).

**TABLE A2:** Morphological and growth properties of *Prolixibacter* isolates

Taxonomic group	*P. denitrificans*			*Prolixibacter* sp.		*P. bellariivorans*
Strain^a^	MIC1‐1^T^	AT004	KGS048	SD074	NT017	JCM 13498^T^
Cell size (µm)	3.4–6.3	1.0–6.5	1.2–3.7	1.2–3.0	1.2–6.0	10.5–12.5^b^
Color of cells	Pink	Pink	Pink	Beige	Beige	Beige
16S rRNA gene sequence identity to						
*P. denitrificans*	−	99.2	99.1	95.6	98.4	97.4
*P. bellariivorans*	97.4	97.4	97.3	97.3	96.5	−
Utilization as the carbon source under aerobic and anaerobic conditions						
Acetate	−	−	−	−	−	−
l‐Lactate	−	−	−	−	−	−
Pyruvate	−	−	−	−	−	−
d‐Glucose	+	+	+	+	+	+

Note

Cells were grown in YSw medium except that Sw medium was used for the screening of carbon sources.

^a^ All strains were rod‐shaped cells, non‐sporulating, non‐motile, negative for Gram staining.

^b^ Data from Holmes et al. (2007).

**TABLE A3:** Corrosion products of Fe^0^ in anaerobic cultures of *Prolixibacter* strains

Strain	Nitrate reducer				Nitrate non‐reducer		–
	MIC 1‐1^T^	AT004	KGS048	SD074	NT017	JCM 13498^T^	Aseptic control
FeCO_3_	+	+	+	+	−	−	−
Fe_3_(PO_4_)_2_	+	+	+	+	−	+	+
FeOFe_2_O_3_	−	−	v	v	−	−	−
FeO(OH)	−	−	v	v	−	−	−

Note

+: detected, v: variably detected, −: not detected. Corrosion products of Fe^0^ were determined in duplicate.

**TABLE A4:** Nitrite‐induced Fe^0^ corrosion

Ratio of nitrate to nitrite	Nitrate (mM)	Nitrite (mM)	Dissolved iron		
			Total iron (mM)	Fe^2+^ (mM)	Fe^3+^ (mM)
NO^3−^: NO^2−^ (10:0)	10	0	0.16 ± 0.01	0.14 ± 0	0.01 ± 0
NO^3−^: NO^2−^ (9:1)	9	1	0.53 ± 0	0.53 ± 0.01	0.01 ± 0
NO^3−^: NO^2−^ (4:1)	8	2	1.2 ± 0.02	1.2 ± 0.04	0.05 ± 0
NO^3−^: NO^2−^ (1:1)	5	5	2.0 ± 0.07	2.0 ± 0.04	0.09 ± 0

Note

The concentrations of ferrous and ferric ions in each culture at day 30 were determined as described previously (Iino, Ito, et al., 2015) except that 2.6 mM ammonium was supplemented to the corrosion test medium. Data represent means and standard deviations (*n* = 3).

**TABLE A5:** The chemical component of SS400 carbon steel and SUS316L stainless steel

Steel grade	Chemical component (%)							
	C	Si	Mn	P	S	Ni	Cr	Mo
SS400	−	−	−	<0.050	<0.050	−	−	−
SUS316L	≦0.030	≦1.00	≦2.00	≦0.045	≦0.030	12.00 ~15.00	16.00 ~18.00	2.00 ~3.00

Note

−: unknown.
